# Bioaccumulation of the Heavy Metal Cadmium and Its Tolerance Mechanisms in Experimental Plant *Suaeda salsa*

**DOI:** 10.3390/ijms26146988

**Published:** 2025-07-21

**Authors:** Qingchao Ge, Tianqian Zhang, Liming Jin, Dazuo Yang, Yang Cui, Huan Zhao, Jie He

**Affiliations:** 1 College of Fisheries and Life Science, Dalian Ocean University, Dalian 116023, China; 18769373351@163.com (Q.G.); dzyang@dlou.edu.cn (D.Y.); yezimeixinqing@163.com (Y.C.); 2 Key Laboratory of Marine Bio-Resources Restoration and Habitat Reparation in Liaoning Province, Dalian Ocean University, Dalian 116023, China; 15004229956@163.com; 3 College of Marine Science and Environment Engineering, Dalian Ocean University, Dalian 116023, China; 4 College of Life Science, Dalian Minzu University, Dalian 116600, China; jlm@dlnu.edu.cn

**Keywords:** antioxidative enzyme, cadmium content, plant growth, *Suaeda salsa*, subcellular distribution

## Abstract

*Suaeda salsa* is relatively tolerant to cadmium (Cd) contamination. In order to investigate the bioaccumulation and stress responses of *S. salsa* under chronic exposure, we explored the growth, accumulation, and changes in antioxidant enzymes and glutathione (GSH) under different Cd concentrations over a 30-day soil culture experiment. Seedling height and weight in the 13.16 mg/kg Cd group were 13.26 cm and 0.21 g, significantly higher than the control group. Growth was significantly inhibited under high Cd concentration exposure, with a seedling and root length of 9.65 cm and 3.77 cm. The Cd concentration in all tissues was positively related to Cd treatment concentration, with the Cd contents in the roots being higher than in the other tissues. At a subcellular level, Cd was mainly concentrated in the cell walls, organelles, and soluble components within the range of 0.05–8.29, 0.02–2.40 and 0.08–1.35 μg/g, respectively. The accumulation of Cd in the roots tracked its proportion in the cell walls. The malondialdehyde (MDA) content of the plant tissues increased with increasing Cd concentration, indicating that Cd stress caused oxidative damage. The GSH content increased with increasing Cd concentration, with maximum values of 0.515 μmol/g in the stem in the 66.07 mg/kg Cd group. The catalase (CAT), superoxide dismutase (SOD), and peroxidase (POD) activity showed different change trends under Cd exposure. The results in this study could provide useful information on the tolerance mechanism of Cd in *S. salsa*, which provides information for exploiting *S. salsa* as a candidate for phytoremediation of Cd contamination.

## 1. Introduction

Heavy metals (HMs) pollution due to increasing urban, industrial, and agricultural development poses significant threats to human and ecosystem health. Due to its long half-life, easy accumulation, and irreversibility characteristics, cadmium (Cd), is recognized as one of the most hazardous HMs and has been detected in marine environments worldwide. The concentration of seawater Cd near the dockyards in the Bay of Bengal has been measured at 4 μg/L, significantly higher than the average seawater Cd concentration, with the concentration in the dockyard sediments reaching 4.81 μg/g [1]. The Cd concentration in the surface sediment of Akyatan Lagoon in the northeastern Mediterranean Sea ranged between 0.11 mg/kg to 1.42 mg/kg [2]. The Cd concentration in the sediments of Abu Dhabi waters ranged from 0 mg/kg to 4.68 mg/kg [3]. In China, the average Cd content in the surface seawater was 13.692 μg/L, and the highest concentration of Cd was observed in the east of Qinzhou Bay in the South China Sea with a content of 428 μg/L [4]. An investigation of HMs in the Liaohe estuary sediments also found Cd levels significantly exceeding environmental quality standards [5]. To cope with the Cd pollution in marine environments, exploring Cd pollution remediation, especially through phytoremediation, has received more attention.

Cd is not an essential element for plants, and exposure to it can produce reduced growth and oxidative damage, affecting plant development [6]. Many plants have evolved a series of tolerance mechanisms to cope with Cd stress. The first barrier in the detoxification metabolism of plants to Cd stress is the plant root system [7]. The components such as cellulose, hemicellulose, and pectin, and structural polysaccharides with functional groups such as carboxyl and sulfhydryl groups in the cell wall could bind Cd and thus reduce Cd entry into the cell [8]. The regional isolation of Cd by the cell wall in the plant root system plays an important role in coping with the stress of Cd. When Cd enters the cells of plants, chelation by ligands such as metallothionein (MT), plant chelating peptides (PCs), glutathione (GSH), citric acid, oxalic acid, and other small molecules in the cytoplasm and vacuole isolation are a vital detoxification pathway [9]. These ligands chelate Cd irons, thereby neutralizing Cd toxicity and facilitating their sequestration in plant vacuoles, reducing the toxicity or detoxifying effect of heavy metals [10]. When Cd enters the cell, it could also trigger a burst of reactive oxygen species (ROS). In order to reduce the adverse effects of ROS, the antioxidant defense system, including superoxide dismutase (SOD), peroxidase (POD), and guaiacol peroxidase (GPX), is activated and the production of non-enzymatic antioxidants, including GSH, is increased [6,11,12]. Above all, a multifaceted defense strategy in plants is activated to reduce damage from Cd stress, which includes exclusion, chelation, transportation, and compartmentalization [13,14].

In recent years, increasing attention has been paid to hyperaccumulator plant species such as *Arabidopsis halleri* and *Solanum nigrum*, etc. [15,16]. However, some of hyperaccumulator plants are difficult to grow in a saline environment [17]. Compared with the hyperaccumulator plants, some halophytes such as *Spartina argentinensis* and *Suaeda salsa* exhibited high tolerance to HMs [17,18]. Clarification of the morphological responses to HMs and the physiological characteristics of those halophytes is vital for an understanding of the mechanisms of metal detoxification, which provides a breakthrough for using halophytes as candidates to remediate saline soils with HMs pollution.

*S. salsa* is an annual herb in the family Chenopodiaceae. It is one of the dominant species in estuarine wetlands in northern China, especially in the Shuangtai estuary in Liaoning Province, which is at high risk of Cd pollution caused by terrigenous wastewater from industrial petrochemical activities [19,20]. Previous studies reported that *S. salsa* could accumulate HMs such as copper (Cu), lead (Pb), and Cd [17,21]. It was found that the bio-accumulation coefficient of *S. salsa* for Cd is 1.87, with a maximum Cd enrichment factor of 64.32, indicating its high heavy metal accumulation potential and making it a candidate for the phytoremediation of heavy metal pollution [17,22]. On a molecular level, the gene expression of glutathione S-transferase (GST) and catalase (CAT) was induced by 10 and 50 μg/L Cd exposure [23], and the significant metabolic changes in amino acids (valine etc.), carbohydrates (glucose etc.), and intermediates of the tricarboxylic acid cycle were observed in *S. salsa* under Cd exposure [24]. The bioaccumulation characteristic and physiological response of *S. salsa* to Cd stress have been widely studied, but most studies have been carried out using hydroponic experiments, which are not the natural growth conditions of *S. salsa*. In this study we examined growth indicators and the subcellular compartment distribution of Cd, as well as its antioxidant enzyme and non-protein thiol activities in soil culture experiments at various levels of Cd contamination over one month of soil cultivation. Our aim was to clarify the bioaccumulation characteristics and the tolerance mechanism of *S. salsa* under chronic Cd stress in relatively natural conditions, which will provide useful information for using *S. salsa* as a candidate for phytoremediation of Cd contamination in saline soil.

## 2. Results

### 2.1. Effects of Different Cd Concentrations on the Growth of S. salsa

The effects of Cd on the growth of *S. salsa* are shown in Table 1. Seedling height and seedling weight increased in all of the test groups. The seedling heights in the groups exposed to 3.23 mg/kg and 13.16 mg/kg Cd were higher than in the control group. However, exposure to 66.07 mg/kg Cd inhibited the plants’ growth, and the seedling height was 11.06% lower than in the control group, a significant difference. The changes in seedling weight over the trial period were similar to those of seedling height, with the highest appearing in the 13.16 mg/kg Cd group with a value of 0.21 g.

The root length of *S. salsa* in the 3.23 mg/kg Cd group was similar to that in the control group, with no significant difference. However, increasing Cd concentrations inhibited root growth, and the root lengths in the 13.16 mg/kg and 66.07 mg/kg Cd groups were 3.98 cm and 3.77 cm, reductions of 14.59% and 19.09%, respectively. There were no significant differences in root weights between the control group and the Cd-treated groups, although 3.23 mg/kg and 13.16 mg/kg concentrations of Cd exposure induced an increase in root weight, while the highest concentration of Cd exposure inhibited root weight somewhat. The low concentration of Cd promoted growth, but a high concentration of Cd exposure inhibited the growth of *S. salsa*.

### 2.2. Characteristics of Cd Accumulation in S. salsa at Different Cd Concentrations

**Figure 1:**
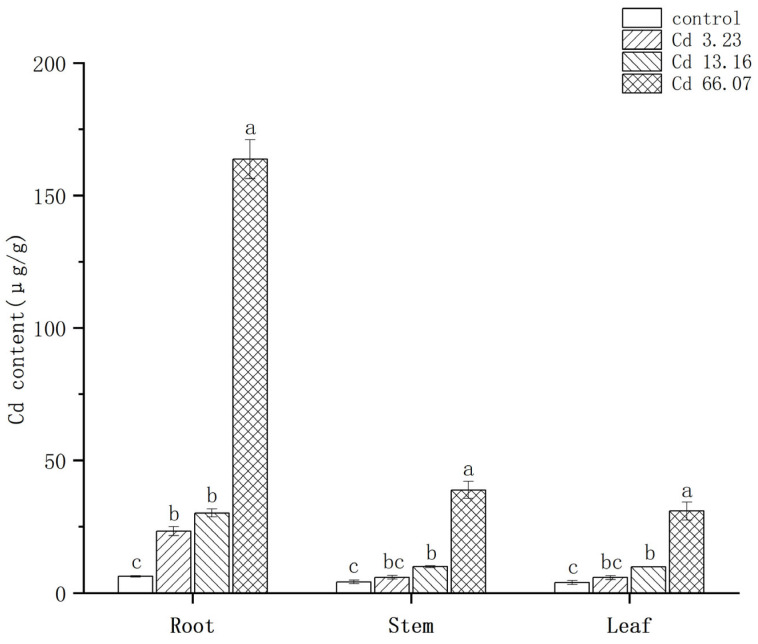
Accumulation characteristics of Cadmium (Cd) in the roots, stems, and leaves of *S. salsa*. The different letters represent the significant difference among different concentrations in each tissue (*p* < 0.05), N = 3.

As shown in Figure 1, the Cd contents of the different tissues of *S. salsa* ranked roots > stems > leaves, and the amount of Cd in the roots was significantly higher than in the stems and leaves. Two-way ANOVA showed that both Cd concentration (F = 1468, *p* < 2 × 10^−16^) and tissues (F = 1022.7, *p* < 2 × 10^−16^) have a significant effect on Cd contents in *S. salsa*, and there was a significant interaction between Cd concentration and tissues (F = 489.2, *p* < 2 × 10^−16^). The Cd contents of the roots in the control, 3.23 mg/kg, 13.16 mg/kg, and 66.07 mg/kg Cd groups were 6.33, 23.37, 30.13, and 163.73 μg/g, respectively. The Cd contents of the stems in these groups were 4.23, 5.97, 9.97, and 38.87 μg/g, respectively. The Cd contents of the leaves were the lowest, with values of 3.97, 5.87, 9.87, and 30.90 μg/g, respectively. The Cd contents of all the tissues were positively related to Cd treatment concentration, and the Cd contents of all the tissues in the 66.07 mg/kg Cd group were significantly higher than in the other groups (*p* < 0.05). The accumulation of Cd in *S*. *salsa* exhibited a tissue-specific expression and was positively related to the Cd concentration.

The bioconcentration factor (BCF) and translocation factor (TF) were evaluated, and the results are shown in Table 2. BCF decreased with an increasing concentration of Cd, with the values of 16.40, 4.73, 1.59, and 1.12 in the control, 3.23, 13.16, and 66.07 mg/kg Cd groups, respectively. TF showed a similar trend to that of BCF. TF in the control group was the highest with a value of 1.29, and TF in the 13.16 mg/kg Cd group was a little higher than that in the 3.23 mg/kg Cd group, with values of 0.66 and 0.51, respectively. TF in the 66.07 mg/kg Cd group was the lowest with a value of 0.43.

### 2.3. Subcellular Distribution of Cd in S. salsa

**Figure 2:**
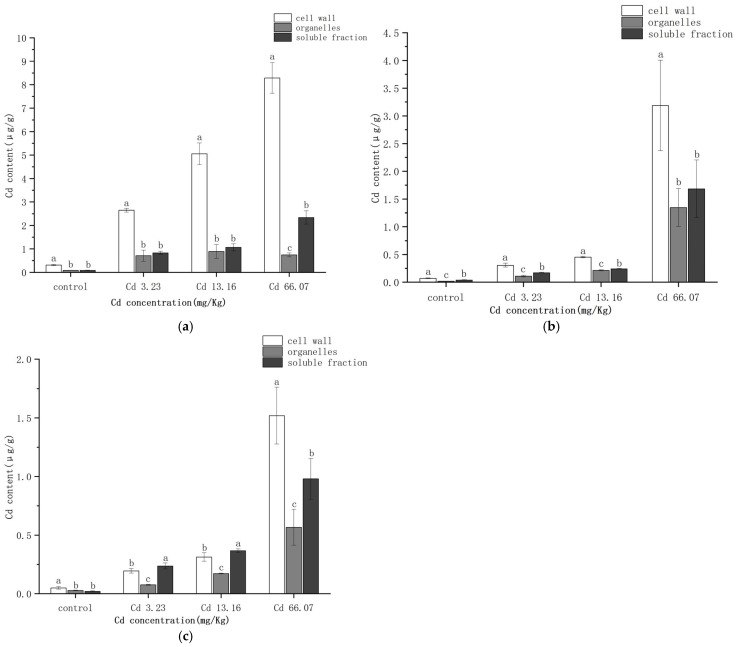
Cd subcellular distribution in the roots (**a**), stems (**b**), and leaves (**c**) of *S.salsa*. The different letters represent the significant difference among various subcellular fractions (*p* < 0.05), N = 3.

The distribution of Cd at the subcellular level in the various *S. salsa* tissues under the different Cd concentrations is shown in Figure 2. Cd was distributed in the cell wall, organelles, and soluble fraction of all tissues. The majority of the Cd was bound to the cell wall (0.05–8.29 μg/g), followed by the soluble fraction (0.02–2.40 μg/g), and then the organelles fraction (0.08–1.35 μg/g).

**Figure 3:**
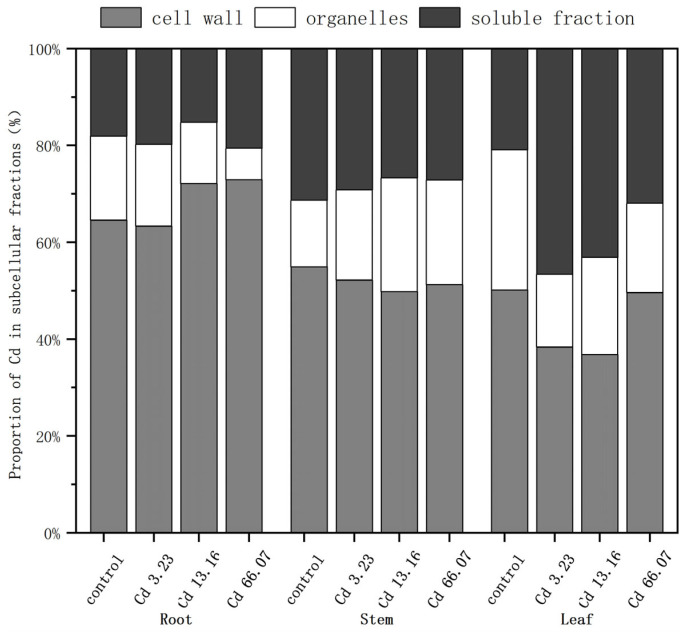
Subcellular proportion of Cd in different tissues of *S. salsa.*

The subcellular distribution of Cd in the roots followed the pattern (Figure 3) cell wall > organelles/soluble fraction. In the roots, Cd was mainly distributed in the cell wall with the proportion of Cd in the cell wall under the different increasing Cd exposure levels of 64.57%, 63.31%, 72.12%, and 72.90%. The proportion of Cd in the cell wall increased markedly with increasing Cd concentration, while the proportion of Cd in the organelles decreased.

The overall trend in subcellular distribution in the stems ranked cell wall > soluble fraction > organelles. The proportion of Cd in the cell wall and soluble fraction decreased with increasing Cd concentration, while the proportion in the organelles increased.

In the leaves of *S. salsa*, Cd was primarily distributed in the cell wall and soluble fraction. In the control group, the distribution trend was ranked as cell wall > organelles > soluble fraction. In the other treatment groups, the proportion in the soluble fraction was clearly higher, and in the 3.23 mg/kg and 13.16 mg/kg Cd treatment groups the ranking was soluble fraction > cell wall > organelles.

Above all, Cd was mainly distributed in the cell wall of *S. salsa*, and the proportion of Cd in the cell wall in the roots changed differently from that in the stems and leaves under an increasing Cd concentration.

### 2.4. Changes in MDA Content and Anti-Oxidative Enzyme Activities in S. salsa

**Figure 4:**
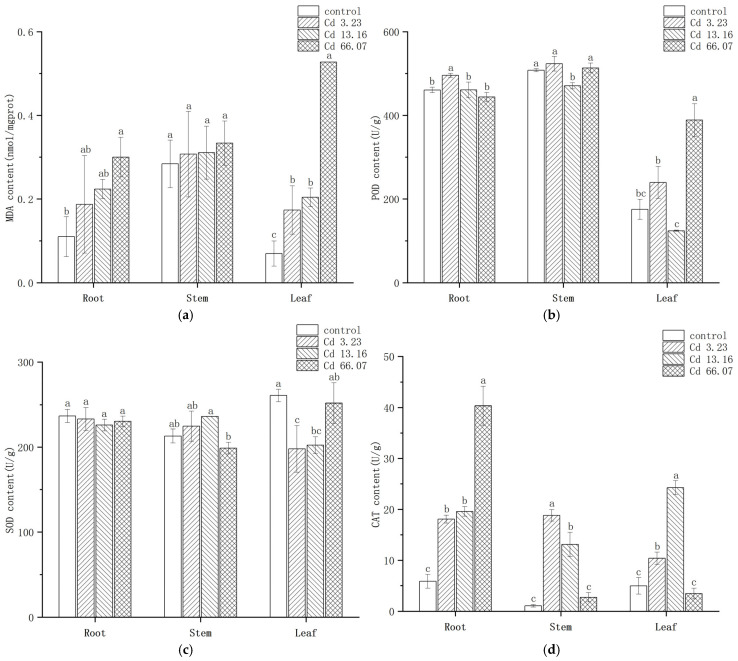
The change of antioxidative enzyme in *S. salsa* under Cd exposure. (**a**) represents the change in malondialdehyde (MDA) Content; (**b**) represents the change of peroxidase (POD) activity; (**c**) represents the change of superoxide dismutase (SOD) activity; (**d**) represents the change of catalase (CAT) activity. The different letters represent the significant difference among Cd concentrations in each tissue (*p* < 0.05), N = 3.

The effect of Cd on the MDA content of the various parts of *S. salsa* is shown in Figure 4a. Two-way ANOVA showed that both Cd concentration (F = 23.484, *p* = 2.55 × 10^−7^) and tissues (F = 8.995, *p* = 0.00122) had a significant effect on MDA content, and there was a significant interaction between Cd concentration and tissues (F = 7.002, *p* = 0.00021). In the roots, the MDA contents in the 3.23 mg/kg and 13.16 mg/kg Cd groups were elevated but not significantly different from the control group (*p* > 0.05). The MDA content in the 66.07 mg/kg Cd group was 1.72 times higher than in the control group, a significant difference. In the stems, there were no significant differences in MDA content among the different Cd concentration treatment groups. In the leaves, there was a significant increase in MDA content in the Cd treatment groups, being 2.51, 2.34, and 7.54 times higher than in the control group, the differences being significant. The MDA content in all tissues increased with the increment of Cd concentration, with the maximum value appearing at 66.07 mg/kg group.

The effect of Cd on POD activity in *S. salsa* is shown in Figure 4b. Two-way ANOVA showed that both Cd concentration (F = 42.06, *p* = 1.03 × 10^−9^) and tissues (F = 674.2, *p* < 2 × 10^−16^) had a significant effect on POD activity, and there was a significant interaction between Cd concentration and tissues (F = 33.98, *p* = 1.38 × 10^−10^). POD activity was induced by 3.23 mg/kg Cd exposure in all tissues of *S. salsa* but was inhibited by 13.16 mg/kg Cd exposure. In the 66.07 mg/kg Cd group, POD activity in the roots was inhibited but increased in the leaves, where it was significantly higher by 2.1 times compared with the control group. The change of POD activity in *S. salsa* in different tissues was various.

The effect of Cd on SOD activity in *S. salsa* is shown in Figure 4c. Two-way ANOVA showed that only Cd concentration (F = 3.125, *p* = 0.045) had a significant effect on SOD activity, but there was a significant interaction between Cd concentration and tissues (F = 9.283, *p* = 2.64 × 10^−5^). SOD activities in the roots and leaves were inhibited and then increasingly induced with increasing Cd concentration. However, SOD activity in the stems was induced at low Cd exposure concentrations but inhibited in the 66.07 mg/kg Cd treatment. SOD activity was highest in the stems in the 13.16 mg/kg Cd group, with a 10.89% increase compared with the control group. Except for the stems, the SOD activity in the roots and leaves was inhibited.

The effect of Cd on CAT activity in *S. salsa* is shown in Figure 4d. Two-way ANOVA showed that both Cd concentration (F = 141.6, *p* = 2.15 × 10^−15^) and tissues (F = 183.4, *p* = 2.88 × 10^−15^) had a significant effect on CAT activity, but there was a significant interaction between Cd concentration and tissues (F = 128.6, *p* < 2 × 10^−16^). CAT activity in the roots increased significantly with increasing Cd treatment concentration and reached its maximum value in the 66.07 mg/kg Cd group, being 6.87 times higher than in the control group. However, CAT activity in the stems and leaves increased and then decreased with increasing Cd treatment concentration. The maximum value in the stems, 18.82 U/g, appeared in the 3.23 mg/kg Cd group, being 17.32 times higher than in the control group. The highest CAT value in the leaves, 24.24 U/g, was in the 13.16 mg/kg Cd group, being 4.87 times higher than in the control group. CAT activity in *S. salsa* was significantly induced in the low concentration Cd exposure.

### 2.5. Changes in GSH Contents in S. salsa Under Cd Exposure

**Figure 5:**
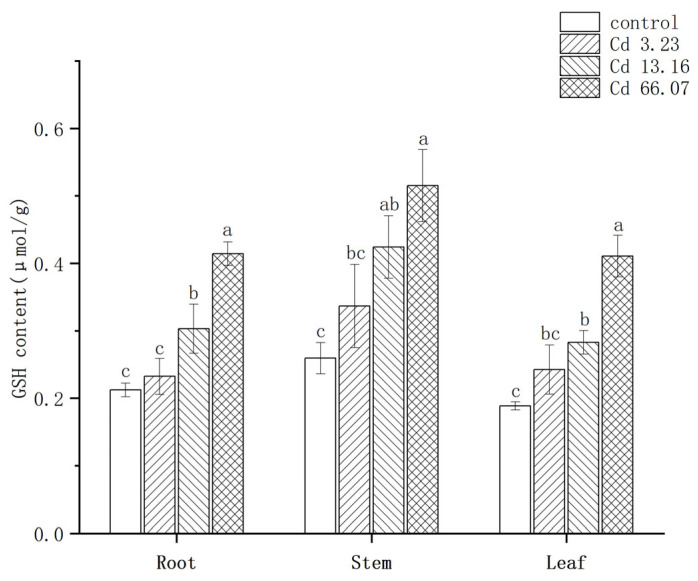
The change of glutathione (GSH) content in *S. salsa* under Cd exposure. The different letters represent the significant difference among concentrations (*p* < 0.05), N = 3.

Figure 5 shows the changes in GSH contents under Cd exposure. Two-way ANOVA showed that both Cd concentration (F = 71.839, *p* = 3.93 × 10^−12^) and tissues (F = 32.224, *p* = 1.59 × 10^−7^) had a significant effect on GSH content, but there was not a significant interaction between Cd concentration and tissues (F = 0.891, *p* = 0.517). The GSH contents of the roots in the 13.16 mg/kg and 66.07 mg/kg Cd groups were significantly higher than in the control group, with increases of 42.86% and 95.23%, respectively. The same upward trend was observed in the stems and leaves with increasing Cd treatment concentration, with maximum values of 0.515 μmol/g in the stem and 0.411 μmol/g in the leaves in the 66.07 mg/kg Cd group. The GSH contents were clearly positively related to Cd concentration in *S. salsa* and were slightly higher in the stems than in other tissues.

### 2.6. The Principal Component Analysis (PCA)

**Figure 6:**
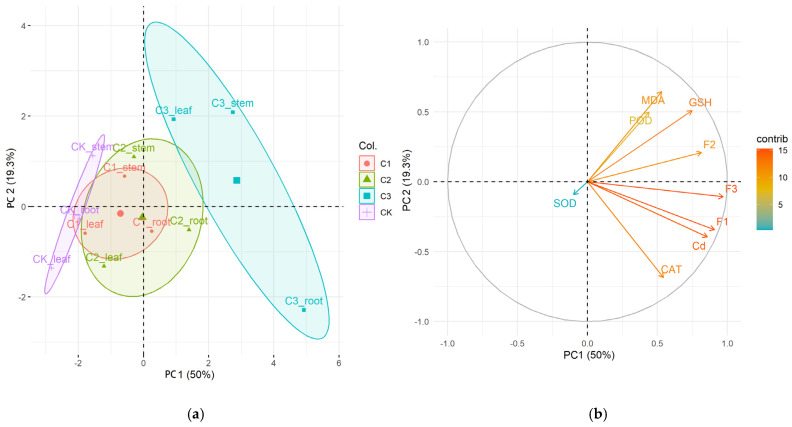
The score and loading of principal component analysis (PCA) on the set of plant morphological data under Cd exposure. (**a**) represents the correlation among variables along two PCA axes (PC1 × PC2); (**b**) represents ordination of case along two PCA axes (PC1 × PC2).

A correlation matrix was established to detect the cluster of cases (Figure 6a). In Figure 6a the area of the control group (peak circle) is to the left, and the area of all Cd concentration groups is separated from the control group, which suggests significant differences between the Cd treatments and the control. The 66.07 mg/kg Cd group was clearly separated from the 3.23 mg/kg and 13.16 mg/kg Cd groups. A correlation matrix of all parameters was performed to detect the relationship between responses to different Cd exposures (Figure 6b). The arrow of the SOD pointed to the lower left, which differed from most other variables. It suggested that SOD may have a weak correlation with other parameters. In contrast, the arrows of MDA, POD, GSH, CAT, subcellular distribution, and Cd contents were closely aligned, indicating strong potential correlations among these variables. Additionally, the Cd contents and subcellular distribution contributed mostly to principal component 1, whereas oxidative stress indicators such as MDA, GSH, and POD contributed mostly to principal component 2.

## 3. Discussion

### 3.1. Influence of Cd Stress on S. salsa Growth

Cd is not considered an essential element for plant growth and development, and its toxicity can induce growth abnormalities in many plants [14,25]. The inhibitory effects of Cd on plant growth have been reported in many plants such as pea (*Pisum sativum*) [26] and *Brassica napus* [27]. In this study, the root length, root weight, seedling height, and seedling weight were inhibited under 66.07 mg/kg Cd exposure, showing the toxic effect of high Cd concentration on *S. salsa*. However, the seedling height and seedling weight of *S. salsa* were significantly increased at low Cd concentrations (3.23 and 13.16 mg/kg) compared with the control group (*p* < 0.05). This phenomenon of ‘low promotion and high inhibition’ in *S. salsa* has been reported in other plants [28]. In *Bidens pilosa*, it was found that low concentrations of Cd promoted its growth, while growth was inhibited by high Cd concentrations [29]. The positive effect of low Cd concentrations on plant growth may be related to increases in transmembrane potential caused by Cd. The abilities of low-Cd (Huajun 2) and high-Cd (Hanlv) *Brassica chinensis* cultivars to accumulate Cd were compared and showed that low levels of Cd exposure stimulated its growth and high Cd exposure levels inhibited its growth [30]. Hyperpolarization of the root surface plasma membranes could be responsible for the growth stimulation under low Cd exposure, causing an increase in trans-membrane potential and enhanced cation uptake [31].

The root system is the primary target of the toxic effects of HMs in plants. In this study, root length growth was increasingly inhibited in *S. salsa* with increasing levels of Cd concentration. Inhibition of root elongation has been reported to be a distinct symptom of Cd toxicity [32]. In contrast to the root length results, the root weights in the 3.23 mg/kg and 13.16 mg/kg Cd groups were higher than in the control group. These increases in root weights could be related to increases in lateral root density. Cd exposure has been found to inhibit primary root growth and enhance lateral root density in *Arabidopsis* and Rhodes grass (*Chloris gayana*) [33,34]. We also observed an increase in the primary root diameter of *S. salsa* when exposed to Cd. It inferred that the inhibition of root length growth, as well as the enhancement of root diameter, could be attributed to the reduced mitotic division of meristematic cells under Cd exposure, causing enlarged cortical tissues to help strengthen the plant’s resistance to water and solute flow [14]. Therefore, increased root weight in the low- and medium-Cd concentration treatments in this study could lead to enlarged cortical tissues and enhance the tolerance mechanisms of *S. salsa* to Cd exposure.

### 3.2. Accumulation and Distribution of Cd in the Different S. salsa Tissues

Plants absorb and accumulate HMs in the soil through a well-developed root system and transfer them to the aboveground parts through root pressure and leaf transpiration, thus reducing the toxicity and mobility of harmful HMs in the soil [35]. In this study, the Cd levels accumulating in the roots of *S. salsa* were significantly higher than in the stems and leaves, and the Cd contents of all tissues gradually increased with increasing Cd concentration. This suggests that the underground parts of *S. salsa* possess a strong Cd retention capacity, reducing the toxicity of Cd to the plant. The result of this study was similar: plants generally store HMs in their roots. Feng et al. found that Cd accumulation varied significantly among the different tissues in rice (*Oryza* spp.), with the highest concentration in the roots, followed by the stems, leaves, and grains [36]. Jia et al. found that small primrose (*Primula* spp.) also accumulated Cd in the root system to a much higher extent than in the leaves and petioles [37]. A study of Cd bioaccumulation in the tissues of *S. salsa* growing in sediments on the Daling River estuary in China reported a trend of roots > leaves > stems [22]. A similar trend was observed in the present study. Taken together, these results suggest that root system retention is an important mechanism in the resistance of *S. salsa* to Cd stress. *S. salsa* is a typical leaf succulent halophyte [38], and the greater retention of Cd in the roots can protect the important photosynthetic physiological activities above the ground, which is one of the reasons why *S. salsa* shows stronger Cd tolerance. The absorption and accumulation of Cd from soil to the roots make *S. salsa* a candidate for phytoremediation in the sediment. Planting *S. salsa* to establish a persistent vegetation cover will help remove Cd from the soil. More field research should be conducted in the future.

The subcellular distribution of Cd in plants is an important factor influencing its transport, accumulation, and toxicity profiles [39]. The toxicity of Cd in plants is influenced by its interactions with different subcellular components. The subcellular structure of plants can be categorized into cell walls, organelles, and soluble components (e.g., vesicles and cytoplasm). The present study found that Cd was mainly enriched in the cell wall, followed by the soluble fractions, and to the least extent in the organelles. The Cd concentrations in the cell walls of the roots, stems, and leaves were 63.31–72.90%, 49.78–54.86% and 36.76–50.12%, respectively, indicating that the cell walls in the roots of *S. salsa* seedlings play an important role in detoxification as well as Cd accumulation. The cell walls enrich polysaccharides and proteins with carboxyl, hydroxyl, amino acids, and other functional groups, which provide binding sites for metal ions, immobilizing HMs to the cell wall and reducing their toxicity to other subcellular structures [40]. Cd can also improve pectinase activity and lower the methylated pectin content in the cell wall, and also induce the rearrangement of cell wall pectin, thereby promoting the absorption and accumulation of Cd by the cell wall [41]. Cd concentration in *Potamogeton crispus* was highest in the cell wall (48–69%), followed by the soluble fraction (16–26%) and organelles (15–26%) [42]. The subcellular distribution of Cd in the roots of chili peppers (*Capsicum* spp.) ranked cell wall > soluble fraction > organelle [43], which is consistent with the present study. Our results were the same as the result in the study of Yang et al. [38]. The subcellular distribution of Cd in *S. salsa* under a hydroponic experiment was also in order of cell wall fraction > soluble fraction > organelle fraction. It indicated that the immobilization of Cd by the cell walls in *S. salsa* could be the main Cd detoxification mechanism at the subcellular level.

When Cd permeates the cell membrane and enters the cytoplasm, it stimulates the plant to synthesize chelating peptides (PCs), which form chelates with Cd to form less-toxic complexes. These complexes are stored in vacuoles, thus reducing the damage to the organelles [44]. Consequently, the vacuoles can be regarded as the second barrier leading to heavy metal detoxification. In this study, the Cd concentrations in the soluble fractions of *S. salsa* exposed to Cd were relatively higher than in the control, suggesting a minor role for vacuolar compartmentalization in the detoxification of Cd in *S. salsa*. The subcellular distribution of Cd in the stems and leaves of *S. salsa* also ranked cell wall > soluble fraction > organelle, but the proportions of the soluble fractions in the stems and leaves were higher than in the roots. We hypothesize that cell wall immobilization and vacuolar compartmentalization play prominent roles in the detoxification of Cd in the stems and leaves of *S. salsa*. When the active groups on the cell wall become saturated with HMs due to binding, excess HMs enter the cell, triggering the plant’s secondary defense mechanism. Organelles such as the Golgi apparatus and endoplasmic reticulum secrete membrane-like substances that compartmentalize the metal ions within specific membrane-bound vesicles. This effectively restricts the free diffusion of metal ions within the cell, preventing damage to critical organelles [45]. We are planning further studies to investigate this suggestion.

### 3.3. Oxidative Damage of S. salsa Due to Cd Exposure

MDA is the end product of lipid peroxidation, and its level is one of the most important indicators of the degree of oxidative damage suffered by a plant under heavy metal stress. The accumulation of MDA increases the permeability of the cell membranes, destroys the structure and function of membranes, and leads to cellular metabolic disorders, which can result in more serious physiological damage and even cell death [46]. This study showed that the MDA contents of the roots, stems, and leaves of *S. salsa* increased significantly with increasing levels of Cd stress, indicating that Cd stress had triggered oxidative damage. Wan et al. observed an upward trend in the MDA content of *S. salsa* under Cu and nickel (Ni) stress [47], and this is consistent with the present study, indicating that heavy metal stress could result in oxidative damage to *S. salsa*. In the present study, the MDA content of the roots and stems showed minimal changes under 3.23 mg/kg Cd treatment compared with the control, indicating that this concentration caused relatively slight damage to the membrane system. However, the MDA content increased significantly in the 66.07 mg/kg Cd treatment, especially in the leaves, indicating that the leaves are the main target site for Cd oxidative damage. This result is consistent with the study by Chen et al., which reported that the MDA contents of *Solanum nigrum* increased with increasing Cd concentration [48]. A similar trend in MDA contents under Cd exposure was also observed in cabbage by Yan et al. [49]. Clearly, the accumulation of MDA can effectively reflect the level of membrane lipid peroxidation damage under Cd stress and could be used as a physiological indicator to evaluate the Cd tolerance of plants.

Cd promotes the production of ROS, leading to protein oxidation and DNA damage, so the production of antioxidant enzymes including SOD, CAT, and POD is one pathway to scavenge ROS and maintain normal metabolic processes [6]. In the present study, the POD and CAT activities in all tissues in the 3.23 mg/kg Cd group were induced. The increased antioxidant enzymes activities in *S. salsa* have also been observed by other researchers. The SOD, POD, and CAT activities of *S. salsa* were significantly induced by combined exposure of 20 μg/L Hg and 500 mM NaCl [50]. Under low concentrations of Pb exposure (100 mg/kg and 200 mg/kg), the SOD, POD, and CAT activities in *S. heteroptera* were induced [20]. Regarding the increment of GST, CAT2 gene expression in *S. salsa* was also observed after two weeks of exposure under 50 μg/L Cd [23]. The increment of POD and CAT activities in this study cooperated to effectively resist the oxidative stress, which was also proved by the increasing content of MDA. The activities of those two enzymes were depressed in the 66.07 mg/kg Cd groups except for the POD activity in the leaves and CAT activity in the stems. The inhibition of antioxidative enzyme activities under high-concentration HMs exposure has been also been reported in *Petroselinum hortense* [51] and *Sedum alfredii* [52]. The reduced enzyme activities may be due to the fact that the accumulation rate of O^2−^ and H_2_O_2_ in *S. salsa* is greater than the scavenging rate of antioxidant enzymes, so the continuous accumulation of ROS exacerbates the peroxidation of membrane lipids, which was consist with the gradual increasing contents of MDA in this study. Compared to other enzymes, the activities of SOD did not exhibit significant change in the stems and roots but was inhibited in the leaves, which was different with the change in POD and CAT activities. The differential changes in SOD, POD, and CAT activities observed in this study could be related to functional differences in the antioxidant enzymes. SOD is the primary enzyme scavenging free radicals in organisms and catalyzes the conversion of superoxide anions (O_2_^−^) into hydrogen peroxide (H_2_O_2_) and oxygen (O_2_). CAT and POD can catalyze H_2_O_2_ into water (H_2_O) and O_2_ [9].

GSH is a sulfhydryl (SH)-containing, low-molecular-weight tripeptide, synthesized through the condensation of glutamate, cysteine, and glycine. It has been suggested that GSH could not only directly neutralize ROS through chemical conjugation but also bind directly with Cd to form non-toxic complexes such as Cd-GS2 [6,53]. In this study, GSH content exhibited a dose-dependent increase with increasing Cd concentration, which is similar to the results in *Brassica napus* [54] and *B. chinensis* [55]. The positive relationship between the change of GSH and the Cd concentration in this study demonstrate that chelation by GSH to Cd could be main detoxification mechanism for GSH in *S. salsa*.

## 4. Materials and Methods

### 4.1. Plant Cultivation and Treatment

Soil was collected from Heishijiao beach (38.8755° N, 121.5656° E) in Dalian, China, sieved to remove impurities, and then dried at 60 °C before use. Three test Cd concentrations were established, according to the marine sediment quality standard (GB18668-2002) [56], 2.5 mg/kg, 12.5 mg/kg, and 62.5 mg/kg, with three replicates for each group. A control group was also established without added Cd. A stock Cd solution was prepared by dissolving 2.03 g of cadmium chloride (CdCl_2_) in deionized water to make a final concentration of 1 mg/mL. This stock solution was then serially diluted and sprayed onto the soil while continuously stirring to ensure uniform mixing. The moistened soil was kept indoors for one week after which the actual Cd concentration was determined. The actual concentrations for the control and each Cd concentration were 0.46 ± 0.03 mg/kg, 3.23 ± 0.14 mg/kg, 13.16 ± 0.81 mg/kg, and 66.07 ± 0.14 mg/kg, respectively.

Seeds of *S. salsa* were purchased from Panjin Xinlongwan Aquatic Products Co., Ltd. (Panjin, China) and stored in the dark indoors. They were surface-sterilized using 3% hydrogen peroxide for 10 min and then rinsed with sterile distilled water. The seeds were then soaked in pure water overnight at room temperature for germination. Seeds weighing 10 g were sown in 3 kg of soil with different Cd concentrations in pots (21.5 cm diameter × 15 cm height). Seedlings were grown at 25/20 °C, day/night temperature; 12 h light/12 h dark photoperiod; 70% relative humidity; and a photo-synthetically active radiation of 6000 Lx.

### 4.2. Analysis of Growth Performance of S. salsa

After 30 d, the plants were removed from the soil, and the root surface was washed carefully to remove substrates. The root length, total plant length, and plant biomass increment were measured.

### 4.3. Determination of Total Cd Content and Subcellular Cd Fractions of S. salsa

The fresh plant tissues were weighed and oven-dried at 80 °C to constant weight. The dried samples were homogenized by powdering, and 100 mg of the dry samples were digested with HNO_3_:HClO_4_ (3:1, *v*/*v*). All of the acids were then removed, and 10% HNO_3_ was added to make 10 mL samples for measurement. In order to investigate the accumulation and transport properties of Cd by *S. salsa*, the bioaccumulation factor (BCF) and translocation factor (TF) were calculated using the following formulas:

(1) $$BCF=\frac{Cd\;concentration\;in\;the\;aboveground\;part}{Cd\;concentration\;in\;the\;soil}$$

(2) $$TF=\frac{Cd\;concentration\;in\;the\;aboveground\;part}{Cd\;concentration\;in\;the\;roots}$$

The subcellular fractions in the *S. salsa* roots, stems, and leaves were separated following the methods of previous studies with minor modifications [57,58]. In brief, 0.5 g of plant tissue was selected, cut into pieces, and homogenized at 4 °C with 5 mL of precooled extraction buffer (250 mM sucrose, 1 mM dithiothreitol, 50 mM Tris-HCl buffer, pH 7.5). The homogenate was centrifuged at 1000× *g* for 15 min to obtain the F1 pellet and S1 supernatant (mainly comprising cell wall fragments). The S1 supernatant was then centrifuged at 10,000× *g* for 30 min to obtain the F2 pellet (organelle fraction) and S2 supernatant (soluble cytoplasmic fraction). All of the separated fractions (F1, F2, S2) were evaporated to dryness and digested using HNO_3_:HClO_4_ (5:1, *v*/*v*). The Cd content of each fraction and the total Cd content were determined using a flame atomic absorption spectrometer (AA800, PerkinElmer, Shelton, CT, USA).

### 4.4. Measurement of Antioxidant Enzyme Activities and GSH Content of S. salsa

A 0.5 g plant tissue sample was weighed and added to 5 mL of precooled nonuple normal saline to make a 10% tissue homogenate. The homogenate was centrifuged at 1800× *g* for 15 min at 4 °C, and the supernatant was collected for enzyme assay. Total protein content in the supernatant was determined using the Bradford assay method and a BioRad™ protein assay kit (BioRad, Hercules, CA, USA).

The malondialdehyde (MDA) content was assayed colorimetrically by monitoring the reaction with thiobarbituric acid under acidic conditions at 532 nm. The SOD and catalase (CAT) activities were determined by measuring the superoxide anion radical (O_2_^−^) and H_2_O_2_ concentrations by absorbance at 560 nm and 240 nm, respectively. POD activity was assayed colorimetrically by monitoring the oxidation of guaiacol at 470 nm. The antioxidant enzyme activities were determined by the kits (Jiancheng Biotechnology Co., Ltd., Nanjing, China) following the instruction.

The homogenate was centrifuged at 12,000× *g* for 10 min at 4 °C, and the supernatant was used to determine the GSH levels by monitoring the reaction with dithiobis-2-nitrobenzoic acid at 412 nm using kits developed by Jiancheng Biotechnology Co., Ltd. (Nanjing, China).

### 4.5. Statistical Analysis

All of the data were analyzed using the SPSS v. 20 statistical software package (IBM, Armonk, NY, USA) and presented as mean ± standard error. Data normality and homoscedasticity were evaluated using Levene’s tests. A two-way ANOVA was used to analyze the interaction between Cd concentrations and tissues specialty. If a significant interaction was observed (*p* < 0.05), a single-factor analysis of variance was applied to analyze the statistical differences between the different Cd exposure treatments. The significance level was set at *p* < 0.05. A principal component analysis (PCA) was run on physiological status (Cd content, subcellular distribution, antioxidative enzymes, and GSH) using R software package v.4.3.2.

## 5. Conclusions

In this study, we investigated the physiological responses of the halophyte *S. salsa* to Cd stress. The growth responses of *S. salsa* to varying levels of Cd exposure followed a hormesis pattern of low-dose stimulation and high-dose suppression. Cd was mainly compartmentalized in the root cell walls, indicating that root immobilization and cell wall binding form synergistic physiological barriers against Cd uptake and translocation in *S. salsa*. Metal chelation by thiol-containing compounds such as GSH and antioxidant action by antioxidative enzymes also played critical roles in the heavy metal tolerance of *S. salsa*. These findings provide information for further exploration of the molecular mechanisms of Cd tolerance in *S. salsa*. In the natural environment, *S. salsa* is exposed to osmotic stress, so further studies are necessary to identify the influence of environmentally relevant salinity on the Cd-induced effect in *S. salsa*, which will provide insights into phytoremediation by *S. salsa* in the field.

## Figures and Tables

**Table 1:** Growth parameters of *S. salsa* under different concentrations of Cadmium (Cd).

Cd Concentration (mg/kg)	Seedling Height (cm)	Seedling Weight (g)	Root Length (cm)	Root Weight (g)
Control	11.88 ± 1.34 ^a^	0.14 ± 0.04 ^a^	4.66 ± 0.97 ^a^	0.015 ± 0.005 ^ab^
3.23	12.25 ± 1.00 ^b^	0.16 ± 0.05 ^a^	4.66 ± 0.72 ^a^	0.016 ± 0.005 ^ab^
13.16	13.26 ± 1.18 ^b^	0.21 ± 0.05 ^b^	3.98 ± 0.62 ^b^	0.019 ± 0.005 ^a^
66.07	9.65 ± 1.24 ^c^	0.12 ± 0.04 ^a^	3.77 ± 0.92 ^b^	0.014 ± 0.005 ^b^

Different letters represent significant differences (*p* < 0.05) among Cd concentrations, N = 20.

**Table 2:** BCF and TF of *S. salsa* under different concentrations of Cd exposure.

Cd Concentration (mg/kg)	Bioconcentration Factor (BCF)	Translocation Factor (TF)
Control	16.40	1.29
3.23	4.73	0.51
13.16	1.59	0.66
66.07	1.12	0.43

## Data Availability

The data supporting reported results can be found in the Supplementary Materials.

## Supplementary Materials

The following supporting information can be downloaded at https://www.mdpi.com/article/10.3390/ijms26146988/s1.

## Abbreviations

The following abbreviations are used in this manuscript:

MDA	malondialdehyde
CAT	catalase
POD	peroxidase
SOD	superoxide dismutase
Cd	cadmium
GSH	glutathione
CdCl_2_	cadmium chloride
HNO_3_	nitric acid
HClO_4_	perchloric acid
H_2_O_2_	hydrogen peroxide
HMs	heavy metals
PCA	principal component analysis
GST	glutathione S-transferase
BCF	bioaccumulation factor
TF	translocation factor
